# Assessment of geothermal resource potential in Changbaishan utilizing high-precision gravity-based man-machine interactive inversion technology

**DOI:** 10.3389/fdata.2023.1139918

**Published:** 2023-07-17

**Authors:** Zhi-He Xu, Ji-Yi Jiang, Guan-Wen Gu, Zhen-Jun Sun, Xuan-Kai Jiao, Xing-Guo Niu, Qin Yu

**Affiliations:** ^1^College of Earth Sciences, Institute of Disaster Prevention, Sanhe, China; ^2^Engineering Research Center of Geothermal Resources Development Technology and Equipment, Ministry of Education, Jilin University, Changchun, China; ^3^Hebei Key Laboratory of Earthquake Dynamics, Sanhe, China; ^4^Engineering Research Center of Geothermal Resources Development Technology and Engineering, Ministry of Education, Jilin University, Changchun, China; ^5^Inner Mongolia Non-ferrous Geological Mining Industry, Huhehaote, China

**Keywords:** north of the Tianchi caldera, geothermal energy exploration, large-scale high-precision gravity, two point five man-machine interactive inversion technology, Northeast China

## Abstract

As one of the clean energy sources, geothermal resources have no negative impact in changing the climate. However, the accurate assessment and precise identification of the potential geothermal resource is still complex and dynamic. In this paper, ~2,000 large-scale high-precision gravity survey points are conducted in the north of the Tianchi caldera, Changbaishan. Advanced data processing technologies can provide straightforward information on deep geothermal resources (Hot source, caprock, geothermal reservoir and geothermal migration pathway). Upwards continuation and the technologies decode two dome shaped low and gentle anomalies (−48 × 10^−5^ m/s^2^−65 m/s^2^) and a positive gravity gradient anomaly (0.4 × 10^−7^ m/s^2^−1.6 × 10^−5^ m/s^2^) in large-scale high-precision gravity planar. According to two point five dimensional man-machine interactive inversion technology and the research on petrophysical parameters, the density of the shied-forming basalts in the two orthogonal gravity sections is 2.58 g/cm^3^. The relatively intermediate to high density (2.60–2.75 g/cm^3^) represents geothermal reservoir, and low density (low to 2.58 g/cm^3^) is the geothermal migration pathway. In addition, large-scale high-precision gravity planar with a solution of about 1/50,000 indicate that the north of the Tianchi caldera exits the sedimentary basin and uplift mountain geothermal system.

## 1. Introduction

Low-carbon economy was firstly proposed in the “Energy White Paper” by the United Kingdom of Great Britain and Northern Ireland government in 2003 (UK Department of Trade Industry, 2003). Following that, the world has undergone a rapid transformation from fossil fuel-based energy to clean energy and many countries have committed to achieving net-zero in the near future (French Interministerial Task Force on Climate Change, 2004; US Climate Action Network, 2004; Greater London Authority, 2007). Geothermal resources as one of the low power consuming, low consumption, low emission, and low pollution clean energy sources is playing an important role in energy transitions (Koji et al., 2007; Wang and Huang, 2022; Li et al., 2023). Geophysical methods for geothermal energy exploration mainly incorporate remote sensing, ground penetrating radar, electromagnetic waves and gravity (Harinarayana and Zlotnicki, 2006; Maithya and Fujimitsu, 2019). The first two are mostly concentrated in shallow subsurface geothermal resources, while the last three focus on deep target (Cheryl et al., 2020; Vishal et al., 2023).

**Figure 1:**
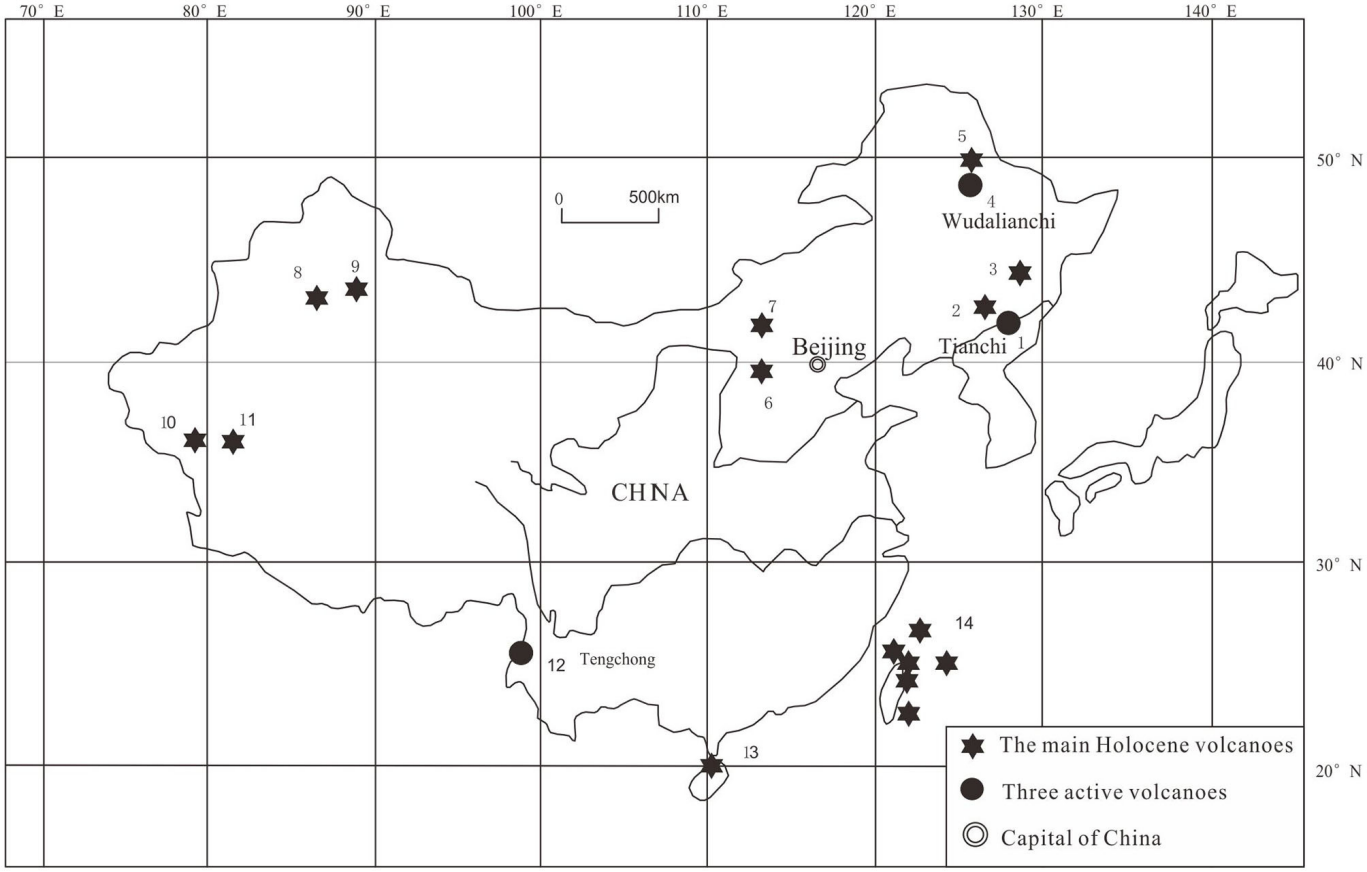
The Holocene volcanoes of China (modified by Wei et al., 2003). 1- Changbaishan Volcano; 2- Longgang Volcano; 3- Jingbohu Volcano; 4- Wudalianchi Volcano; 5- Keluo Volcano clusters; 6- Datong volcano; 7- Honggeertu volcano; 8- Tianshan Volcano; 9- Tianshan Volcano; 10- Turfan Volcano; 11- The Yutian volcano; 12- Tengchong Volcano; 13- Leiqiong Volcano; 14- Datun Volcano.

**Figure 2:**
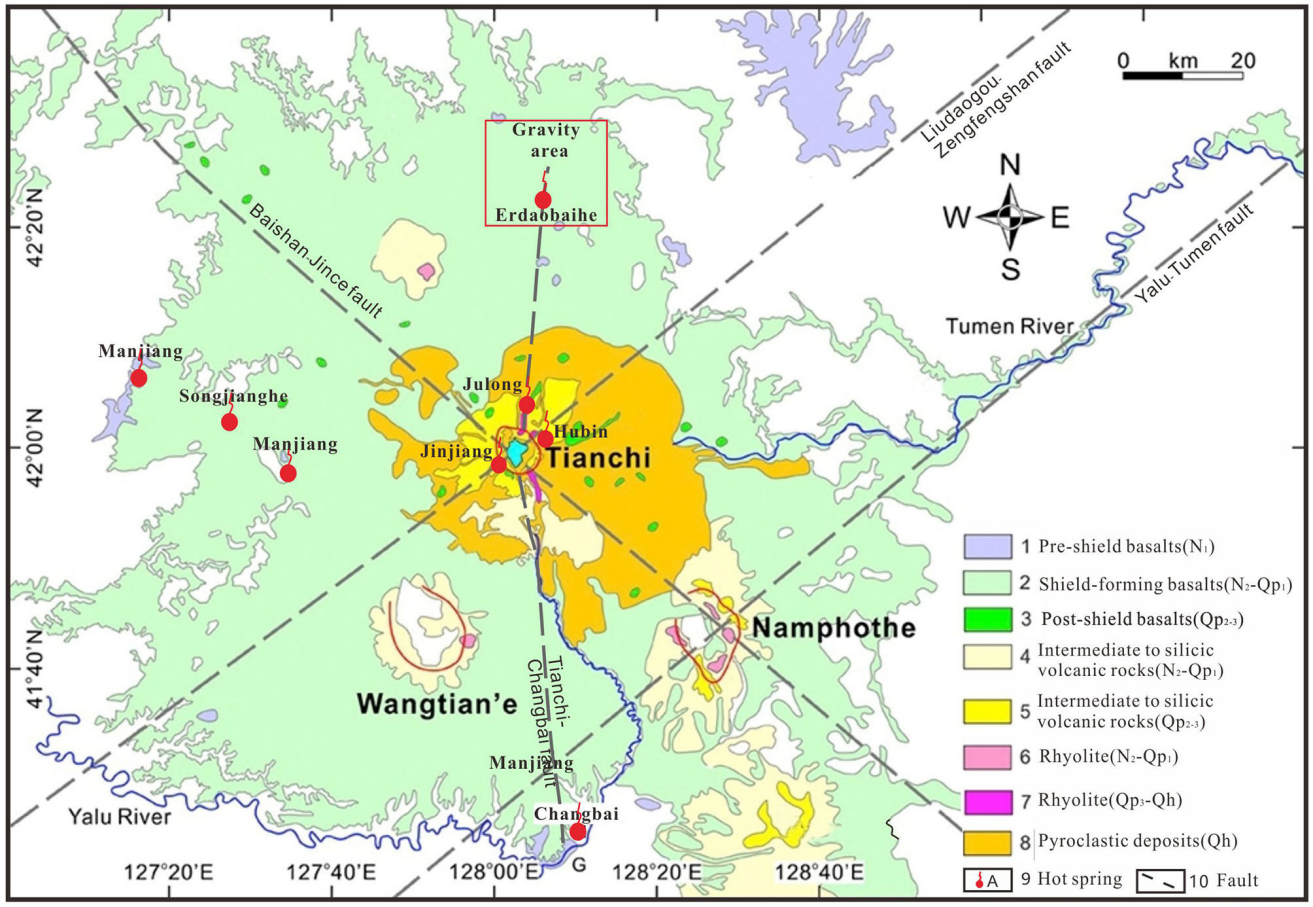
Simplified regional geological map of the Changbaishan Volcano showing the locations of hot springs (modified from Qian et al., 2016).

Changbaishan geothermal field is located in one of the active volcanoes in China (Figure 1) (Cashman and Sparks, 2013; Sun et al., 2017; Xu et al., 2021). The proven and commercially viable geothermal resources have been explored and monitored by remote sensing or ground penetrating radar around Tianchi caldera (Figure 2) (Qian et al., 2016; Tang et al., 2017). However, these shallow subsurface geophysical methods can poorly penetrate the great thick cover of Quaternary Shied-forming basalts to effectively identify and detect the spatial distribution of heat source, geothermal reservoir and the migration pathway in depth. In order to address these issues, use of deep exploration geophysical methods such as gravity, electromagnetic, and seismic exploration which have been used traditionally to detect the spatial distribution of different geological bodies and boundaries can be effective (Gessner et al., 2016; Xu et al., 2019, 2021). Another way is by exploring data-driven solutions, including data processing, modeling, inversion, detection, classification, and so on (Xu et al., 2021).

In this paper, evaluation, potential analyses and types of geothermal resources in north Tianchi caldera, Changbaishan are deciphered by 2,000 large-scale high-precision gravity survey points. In addition, advanced data processing such as upwards continuation, the first vertical derivative, and two point five dimensional man-machine interactive inversion technologies are adopted to precisely portray the spatial distribution of hot source, geothermal reservoir, cap-rock and geothermal migration pathway.

## 2. Geological setting

The Changbaishan Volcanic Group consists of three polygenetic volcanoes: Tianchi, Wangtian'e and Namphothe and numerous Monogenetic volcanoes (Figure 2) (Horn and Schmincke, 2000; Németh and Kereszturi, 2015). The Cenozoic Manjiang formation shied-forming basalt shield covers about 2 × 104 km^2^ area, and the intermediated to silicic volcanic rocks clutter around the Tianchi caldera (Pan et al., 2017; Wei et al., 2017). The Paleoproterozoic stratigraphic units and Archean gneiss are exposed sporadically in the north of the Tianchi caldera (Kimura et al., 2018). There are four major faults: one NW-SE, two NE-SWs, and one N-S trending faults mainly controlling the distribution of geothermal resources (Figure 2) (Zhang et al., 2018).

As the only active volcano in the Changbaishan Volcanic Group, Tianchi caldera is a promising geothermal resources (about 120 hot springs) (Zhang et al., 2018; Xu et al., 2021). Information on the geothermal system types, names, locations, coordinates, temperatures, and water yields are listed in Table 1. According to the developed and utilized geothermal resources, hot springs associated with continuous advection of heat from volcanic activity demonstrate high heat, whereas the non-magmatic type is the low one.

**Table 1:** Typical geothermal hot springs list in Changbaishan.

**Type**	**Name**	**Coordinate**	**Temperature (°)**	**Water yield (m^3^/d)**
Uplift mountain geothermal system	Jinjiang	127°59′33″ 41°56′22″	58–60	578
	Hubin	128°03′45″ 42°01′12″	42–73	<80
	Julong	128°03′30″ 42°02′27″	58–60	6,455
Sedimentary basin geothermal system	Changbai	128°07′00″ 41°25′04″	38–40	346
	Xianrenqiao	127°11′12″ 42°08′56″	45–62	96
	Manjiang	127°35′48″ 41°56′17″	30–31	120
	Songjianghe	127°30′23″ 42°02′27″	25–28	120
	Erdaobaihe	128°06′09″ 42°24′08″	16–20	<50

## 3. Geophysical analytical techniques

### 3.1. Petrophysical parameters

Petrophysical parameters are a bridge between geology and geophysics, and are often used as the foundation for forward modeling and inversion (Eric, 1999; Wang et al., 2015; Xu et al., 2019). Density of about 180 samples was measured by a domestic digital DH-300 density meter with an accuracy of ±0.001 g/cm^3^. Information on the specific number, lithology, and sample locations is listed in Table 2. Samples include all lithology the shied-forming basalt, volcanic rocks, Late Triassic intrusion, Paleoproterozoic stratigraphic units, and Archean gneiss in study area, which help us decipher the gravity anomaly.

**Table 2:** Petrophysical parameters data for different geological units.

**Formation**	**Number**	**Lithology**	**Density (g/m^3^)**	**Average (g/cm^3^)**
Junjianshan	25	Trachybasalt	2.44	2.59
	15	Massive basalt	2.78	
	15	Olivine basalt	2.50	
Guosongzu	30	Volcanic tuff	2.67	2.60
	5	Tuffaceous conglomerate	2.52	
Wangdeshan	25	Dolomitic marble	2.78	2.75
	25	Silicified dolomitic marble	2.72	
	5	Cataclastic marble	2.70	
Archean gneiss	15	Tonalite gneiss	2.73	2.71
	10	Granodioritic gneiss	2.64	
Late Triassic intrusion	5	Medium grained alkali feldspar granite	2.57	2.55
	5	Fine grained alkali feldspar granite	2.53	

The samples from the Late Triassic intrusion exhibited the lowest density. The shied-forming basalt and volcanic rocks were characterized by a low to intermediate density. Archean gneiss showed an intermediate density, while the Paleoproterozoic stratigraphic units were characterized by highest densities. These differences in density allowed the need for geophysical research.

### 3.2. High precision gravity method

A total of 2,000 large-scale high-precision ground-based gravity survey points were collected using a CG-5 type Gravimeter (Scintrex Ltd., Canada) at a scale 1/50,000. The surveyed area was ~400 km^2^ with a density of 4 to 6 measurement points per square kilometer. Subsequently, the data were gridded using the Gauss–Kriging method to provide detailed information of the geological bodies and structures (Han et al., 2020).

Corrections for Free-air and Bouguer anomalies were done using the International Association of Geodesy (1971) 1,967 formula considering 2.67 g/cm^3^ as the density of the middle layer (Morelli et al., 1972; Sundaralingam, 1987; Luo and Yao, 2007; Xu et al., 2022a). Corrections for the gravimetric terrain in near (0–20 m), intermediate (20–2,000 m) and remote areas (2–20 km) were performed using the Trupulse 200 type laser height instrument monitoring data, 1:10,000 scale elevation database and 1:50,000 scale elevation database, respectively. Finally, based on cone-shaped column formula, the topographic correction value of each gravity points are calculated (Laramie and Ralph, 2003; Xu et al., 2022a). The theoretical formula is listed as follows.


(1) $$\begin{array}{l} \Delta {\text{g}}_{T}=2\pi G\rho R\left(\cos i\right)/n \end{array}$$

Where, Δg-Gravity anomaly (m/s^2^), ρ-Density of topographic correction (2.67 g/cm^3^), *R*-Topographic correction radius, *i*-Slope angle, *n*-Azimuth number.

### 3.3. Advanced data processing techniques

Upwards continuation and first vertical derivative are two efficient methods for processing initial gravity data and each has its own merits (Lu et al., 2019; Xu et al., 2022b). The advantage of upwards continuation is that it can segment the deep Bouguer anomalies from the background. Various heights in the range of 200–1,000 m were adopted to highlight the various depth anomalies. In contrast, the advantage of the first vertical derivative is that, it enhances the local anomalies caused by subsurface geological bodies (Cooper and Cowan, 2004). The present study maximized the advantages of both methods for processing the original gravity data.

**Figure 3:**
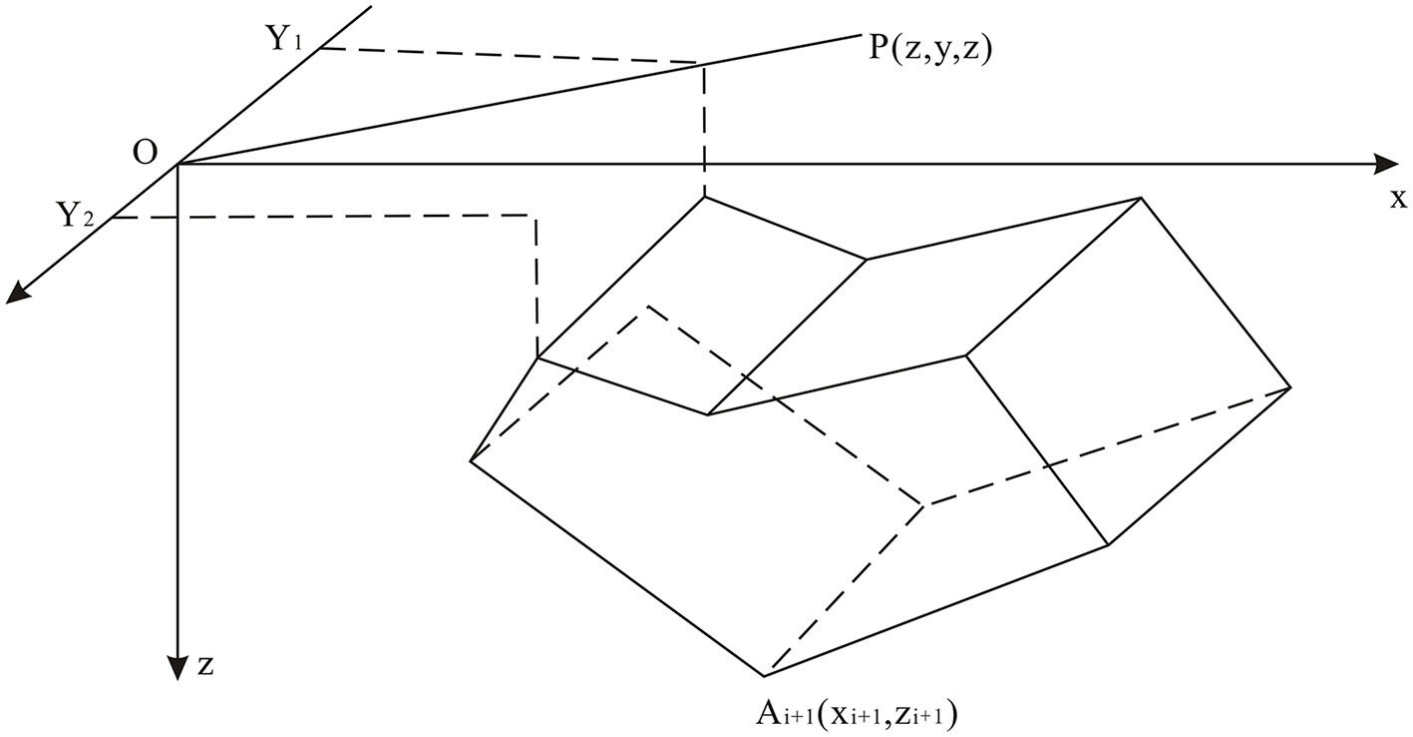
Schematic diagram of the two point five dimensional level prism (modified after Xu et al., 2022a).

Further, two point five dimensional man-machine interactive inversion technology is a perfect compromise between quantitative interpretation of the complicated anomalies and varying the theoretical parameters (Pinto and Casas, 1996). It is used to decipher the four elements of deep geothermal resource (heat source, geothermal reservoir, caprock, and geothermal migration pathway) (Ahumada et al., 2022). The first step for the prediction model was established according to the estimated geological bodies. The theoretical gravity curve was subsequently generated based upon the estimated geometry of the density distribution. The shape of the model is terminated at the least difference between the calculated and measured anomalies (Yao et al., 1998). Finally, hypothetical geological bodies were accomplished by two dimensional interactive inversion. The theoretical formulas and computational model are listed as follows (Figure 3) (Luo and Yao, 2007; Fan et al., 2014).


(2) $$\begin{array}{lll} \Delta \text{g}\left(P\right) & = & G\sigma \overset{N}{\underset{i=1}{\sum }}\cos\varphi_{i}\left[I_{i}\left(Y_{2}\right)-I_{i}\left(Y_{1}\right)\right] \end{array}$$

(3) $$\begin{array}{l} \begin{array}{lll} I_{i}(y) & = & y\ln\;\frac{u_{i+1}+R_{i+1}}{u_{i}+R_{i}}+u_{i+1}\ln\;(R_{i+1}+y)-u_{i}\ln\;(R_{i}+y) \end{array}\\ \begin{array}{ll} \;- & w_{i}(\arctan\frac{u_{i+1}y}{w_{i}+R_{i+1}}-\arctan\frac{u_{i}y}{w_{i}R_{i}}) \end{array} \end{array}$$

(4) $$\begin{array}{lll} u_{i} & = & x_{i}\cos\varphi_{i}+z_{i}\sin\varphi_{i} \end{array}$$

(5) $$\begin{array}{lll} u_{i+1} & = & x_{i+1}\cos\varphi_{i}+z_{i+1}\sin\varphi_{i} \end{array}$$

(6) $$\begin{array}{lll} R_{i} & = & {\left(x_{i}^{2}+y^{2}+z_{i}^{2}\right)}^{1/2} \end{array}$$

(7) $$\begin{array}{lll} R_{i+1} & = & {\left(x_{i+1}^{2}+y^{2}+z_{i+1}^{2}\right)}^{1/2} \end{array}$$

(8) $$\begin{array}{lll} \varphi_{i} & = & -x_{i}\sin\varphi_{i}+z_{i+1}\cos\varphi_{i} \end{array}$$

Where, Δ*g*-gravity anomaly (m/s^2^), *P*-position, *G*-constant value of earth's gravity (N•m^2^/kg^2^), σ-prism density, *i*-prism corner number, *N*-number of prism, *I*-incidence, *y-*Coordinate in *y* direction, *w*_*i*_-Center angle (rad/s).

## 4. Results

### 4.1. Gravity planar results

**Figure 4:**
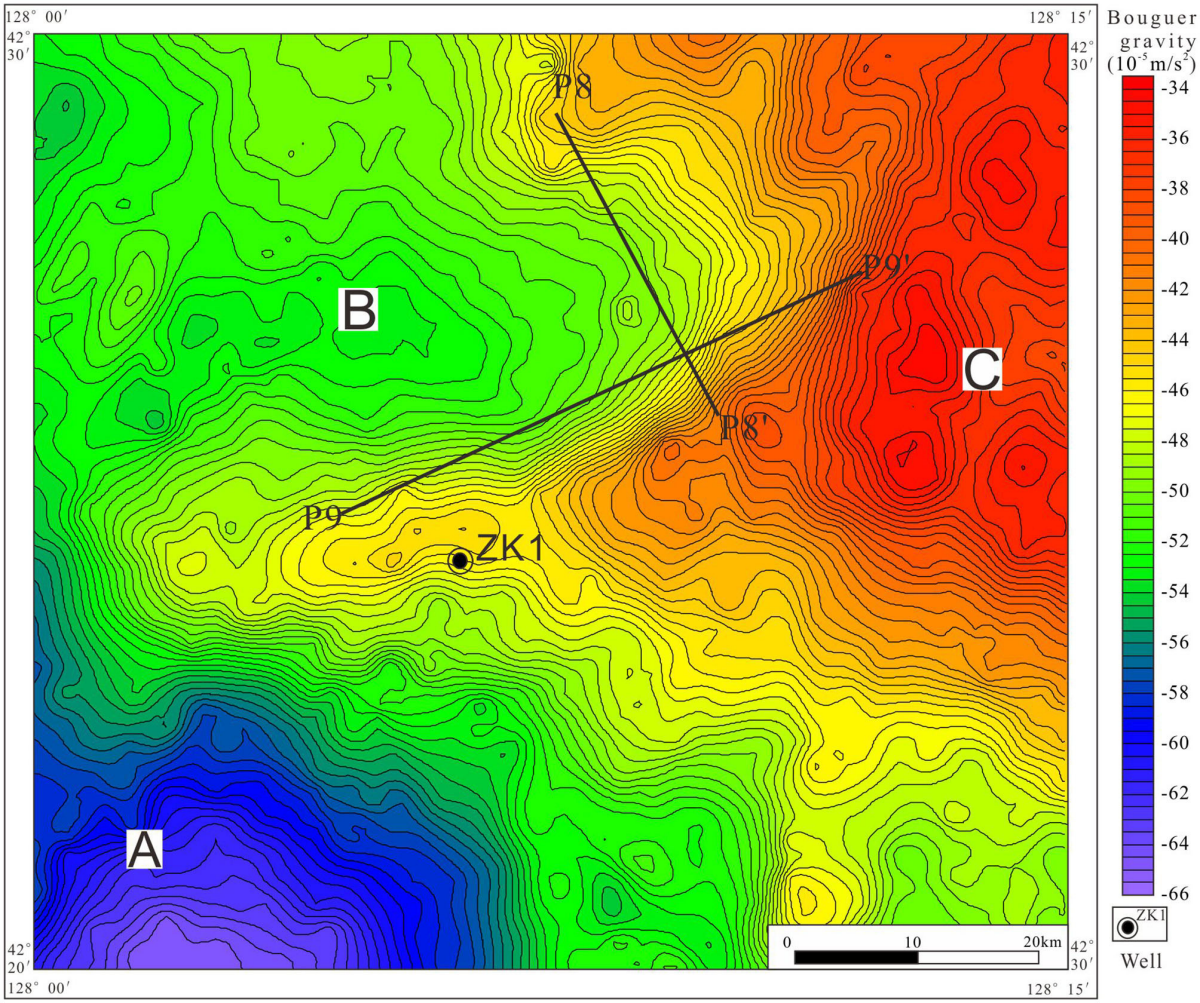
Bouguer anomaly of large-scale high-precision gravity survey points (including the location of P8 and P9 gravity sections). **(A)** Low value area. **(B)** Mid-value area. **(C)** High value area.

The interpretation of gravity data involves i) the division of sub-regions with striking discrepancies in the gravity field, ii) geological inference of Bouguer anomalies, and iii) the determination of the spatial distribution of anomalies (De Castro et al., 2014). Figure 4 presents the distribution of the Bouguer anomalies in the north of Tianchi caldera. The range of remarkable lowest Bouguer anomaly A zone is from −57 × 10^−5^m/s^2^−66 × 10^−5^m/s^2^, the B anomaly zone is from −47 × 10^−5^m/s^2^−56 × 10^−5^m/s^2^, and the C zone is from −34 × 10^−5^m/s^2^−46 × 10^−5^m/s^2^.

These results indicate the existence of density difference in north of Tianchi caldera. According to the descriptions of geological setting and petrophysical parameters in Section Petrophysical parameters, the low anomaly is related to volcaniclastic rocks, and the high anomaly is related to the Paleoproterozoic stratigraphic units or Archean gneiss (Figure 4).

**Figure 5:**
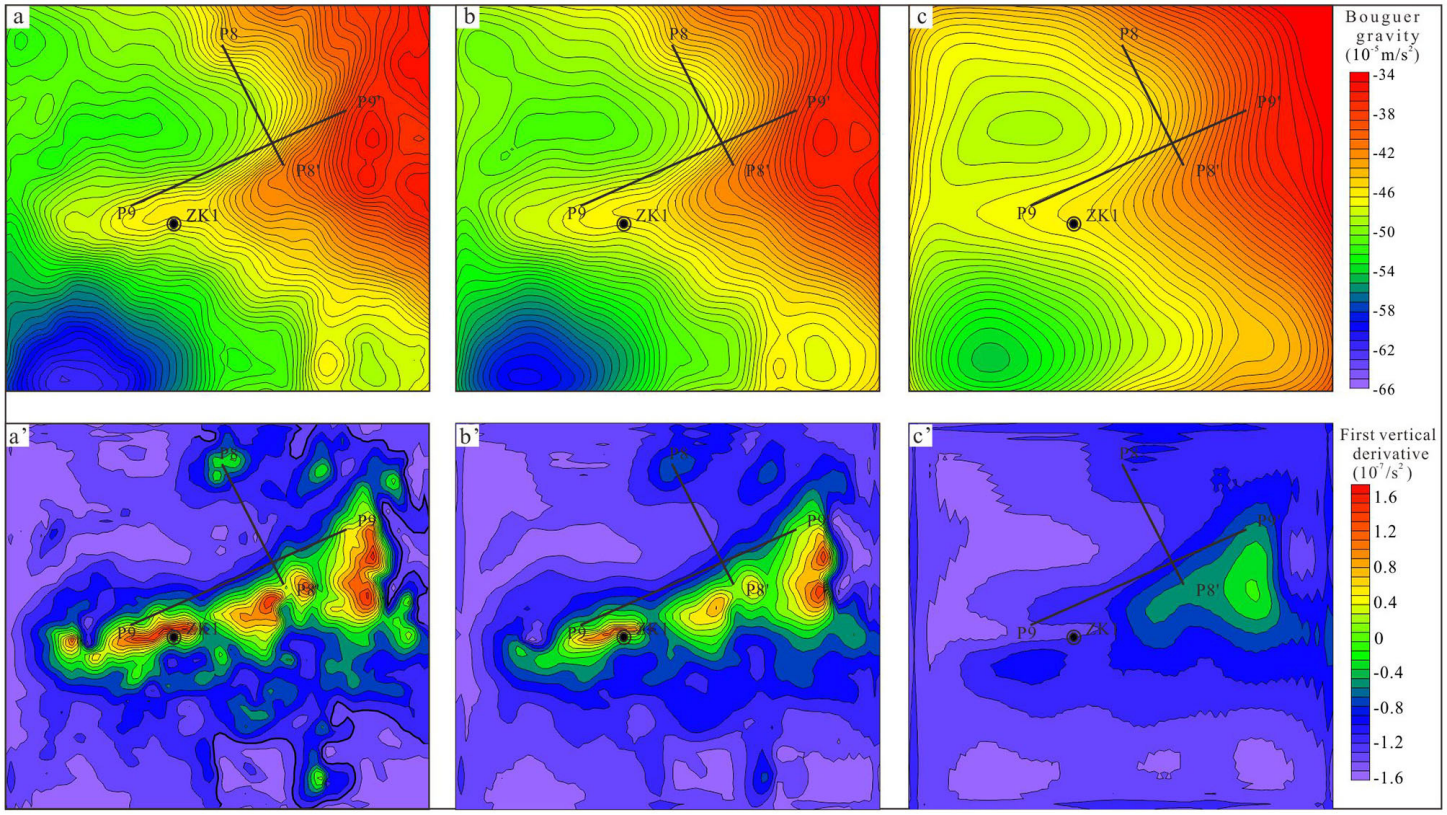
Results of high-precision Bouguer anomaly based on advanced data processing techniques. **(a–c)** The 200 m, 400 m, and 1,000 m upward continuation results; **(a'–c')** the first vertical derivative results of the various upward continuations (200 m, 400 m, and 1,000 m).

However, the boundaries between volcaniclastic rock and Paleoproterozoic stratigraphic units or Archean gneiss are faintly discernible. The results of upwards continuation with different heights (200 m to 1,000 m) indicate that the value of low and high gravity anomaly decreased rapidly with increasing depth, leaving two dome shaped low anomalies (Figures 5a–c). The first vertical derivative results highlight the scope of gravity gradient (Figures 5a'–c').

### 4.2. Gravity sectional results

**Figure 6:**
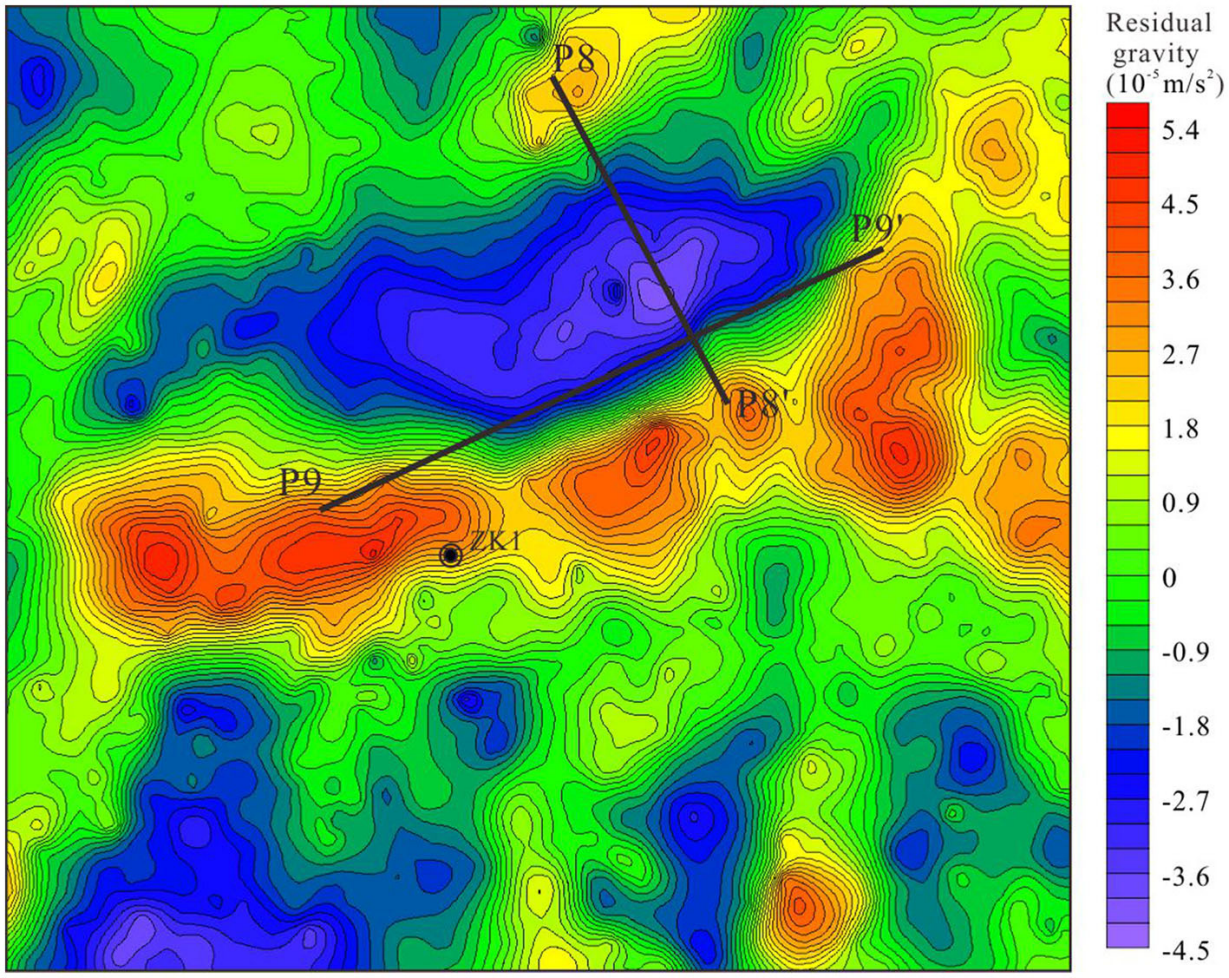
Residual gravity anomaly of large-scale high-precision gravity survey points.

There is a close link between gravity value and geological factors, such as petrophysical properties and geological structures (Yao et al., 1998; Xu et al., 2022b). Residual gravity anomaly has been separated from the Bouguer gravity for carrying out two point five dimensional man-machine interactive inversion technology (Figure 6). Then, two orthogonal gravity sections (P8 and P9) were extracted from the residual gravity anomaly.

**Figure 7:**
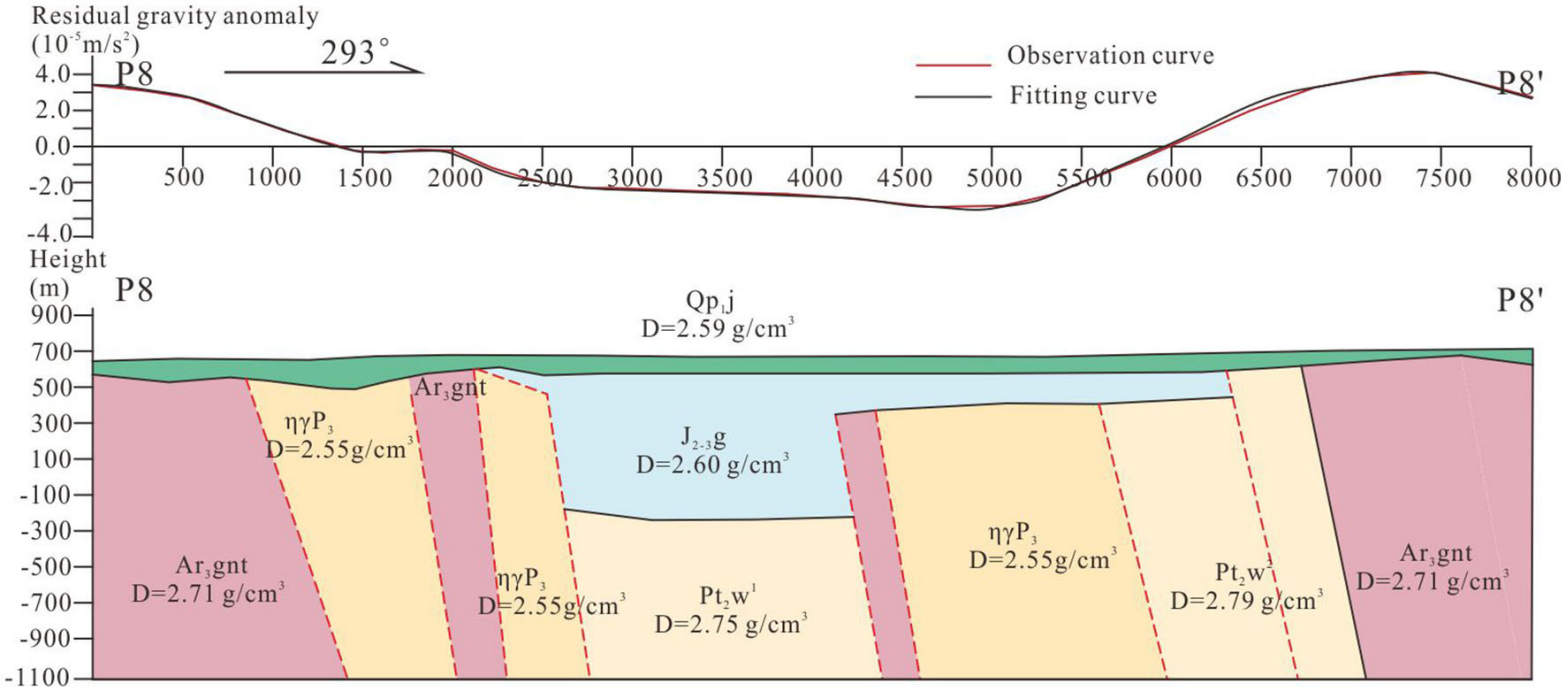
Results of P8 based on two point five dimensional man-machine interactive inversion technology.

**Figure 8:**
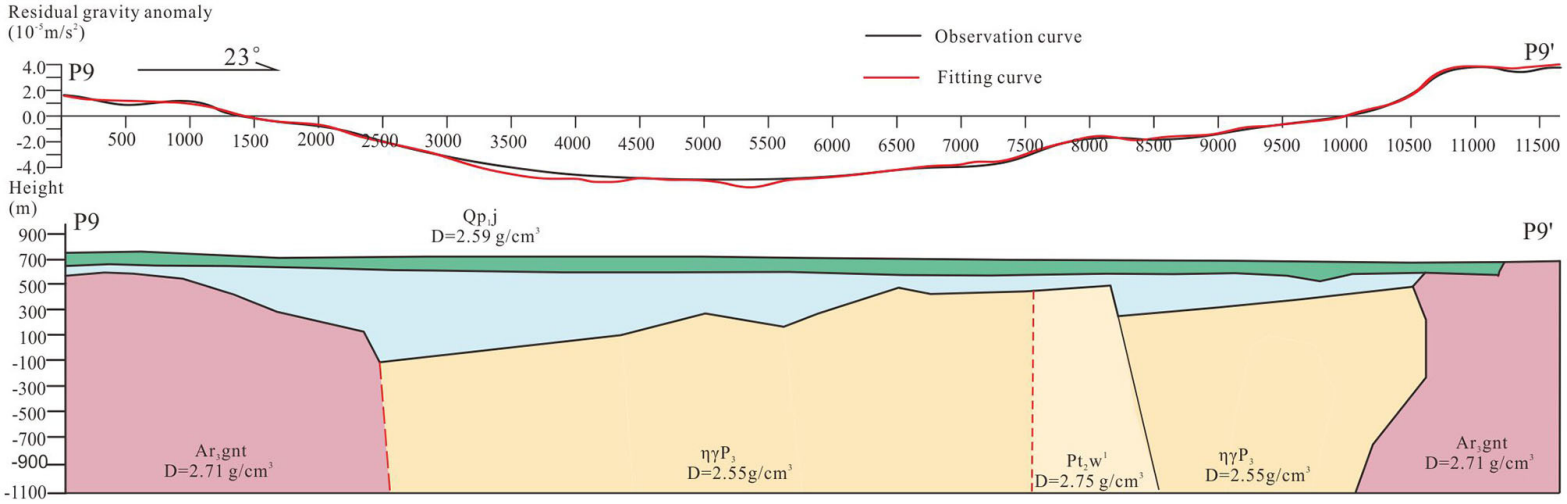
Results of P9 based on two point five dimensional man-machine interactive inversion technology.

P8 and P9 showed a similar trend with a C-type gravity distribution. The gravity value varied from −6 × 10^−5^ m/s^2^−4 × 10^−5^ m/s^2^ (Figures 7, 8). This trend demonstrates that the density in middle are lower than that in both ends and there exits the geological structures crowd zones.

On the basis of surface exposure and statistical distribution in Section Petrophysical parameters, the theoretical gravity curves (red lines) are matching to the field surveyed gravity curves (black lines) and are deducing deep geological bodies and structures (Figure 7). From west to east, the high density geological body (~2.77 g/cm^3^) is the Archean metamorphic crystalline basement, the low ones are Late Permian monzogranite (~2.55 g/cm^3^) and Middle to Late Jurassic pyroclastic rock (~2.60 g/cm^3^), and the high one is Paleoproterozoic marble (~2.79 g/cm^3^). Because of the large porosity, loose cementation and high permeability, the lower pyroclastic rocks are generally regarded as the geothermal reservoir. The gravity gradient zones (sties 2,500 and 4,000) indicate that there exit geological structures which provide migration pathway for deep geothermal resource. In addition, the shied-forming basalt shield (~2.59 g/cm^3^) in the shallow overlying the geothermal reservoir acts as a caprock reducing heat loss function (Figure 8).

## 5. Discussion

### 5.1. Geothermal resource in north Tianchi caldera

As the only active volcano in Changbaishan volcanic field, Tianchi volcano has shown huge potentials in geothermal resources (Zhang et al., 2018). More than 120 hot springs congregate around the Tianchi caldera (Xu et al., 2021). However, due to different control conditions of geothermal resources, uplift mountain or sedimentary basin geothermal systems show different geophysical anomaly (Zhang et al., 2018). Taking the Magmatic type (I_1_ type in uplift mountain geothermal system) as example, continuous advection of heat from Tianchi volcano which provides powerful thermal energy and thermal cycle to directly heat the hydrothermal reservoirs is the hot source (Table 3). The geothermal gradient or radioactive element decay with the geothermal gradient >3.0°C/100 m provides heat for medium to low geothermal resource in the sedimentary basin geothermal system.

**Table 3:** Elements combination of geothermal resources in North Tianchi caldera.

**Geothermal type**	**Hot source**	**Caprock**	**Reservoir**	**Pathway**
Uplift mountain geothermal system	Volcano	Basalts	Pyroclastic rock	Deep fault
	Intrusion			
	Melting rock			
Sedimentary basin geothermal system	Geothermal gradient	Basalts	Cataclastic marble	No required
	Radioactive element			

**Figure 9:**
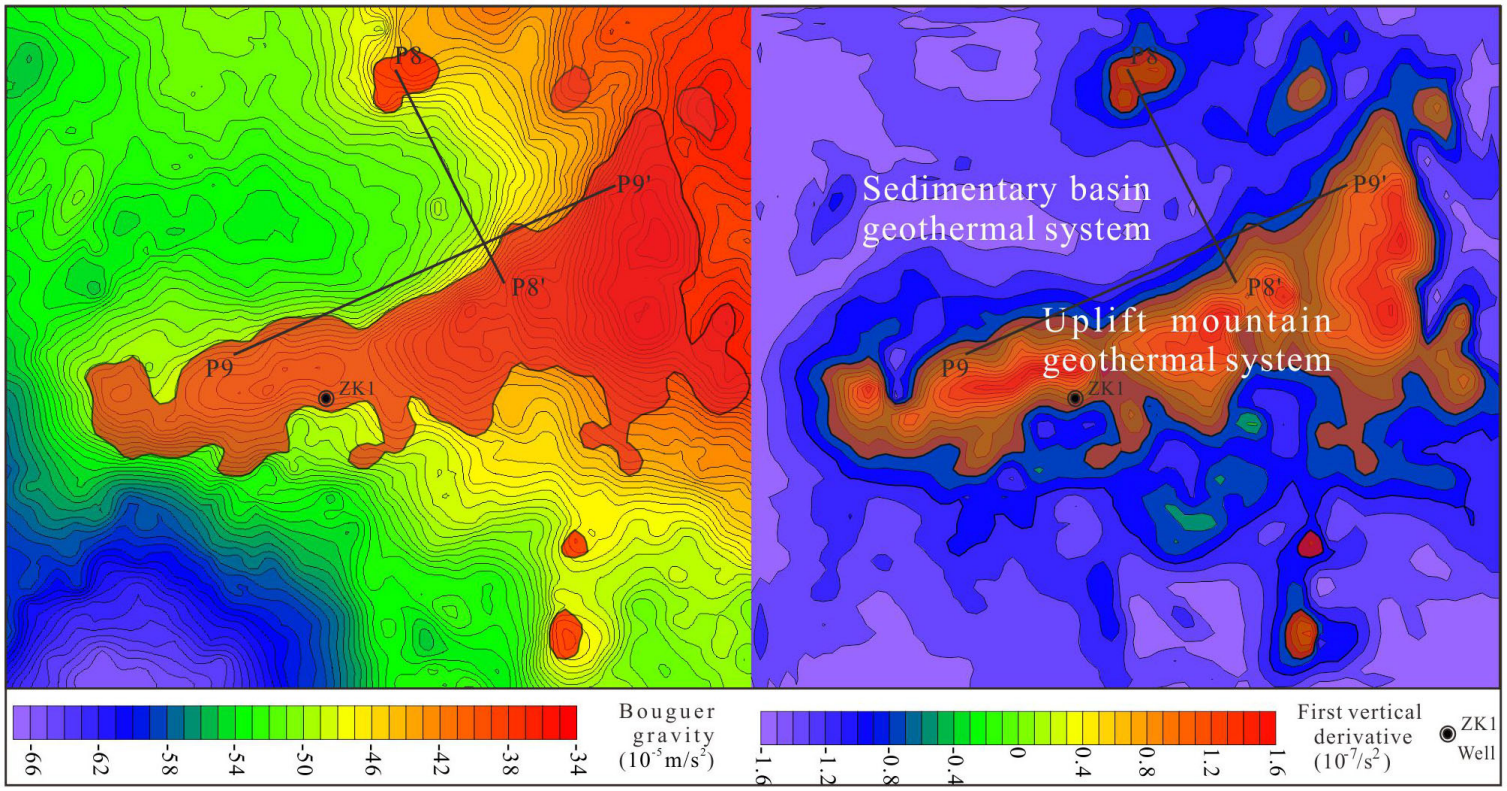
The distribution of sedimentary basin and uplift mountain geothermal system in North Tianchi caldera.

**Figure 10:**
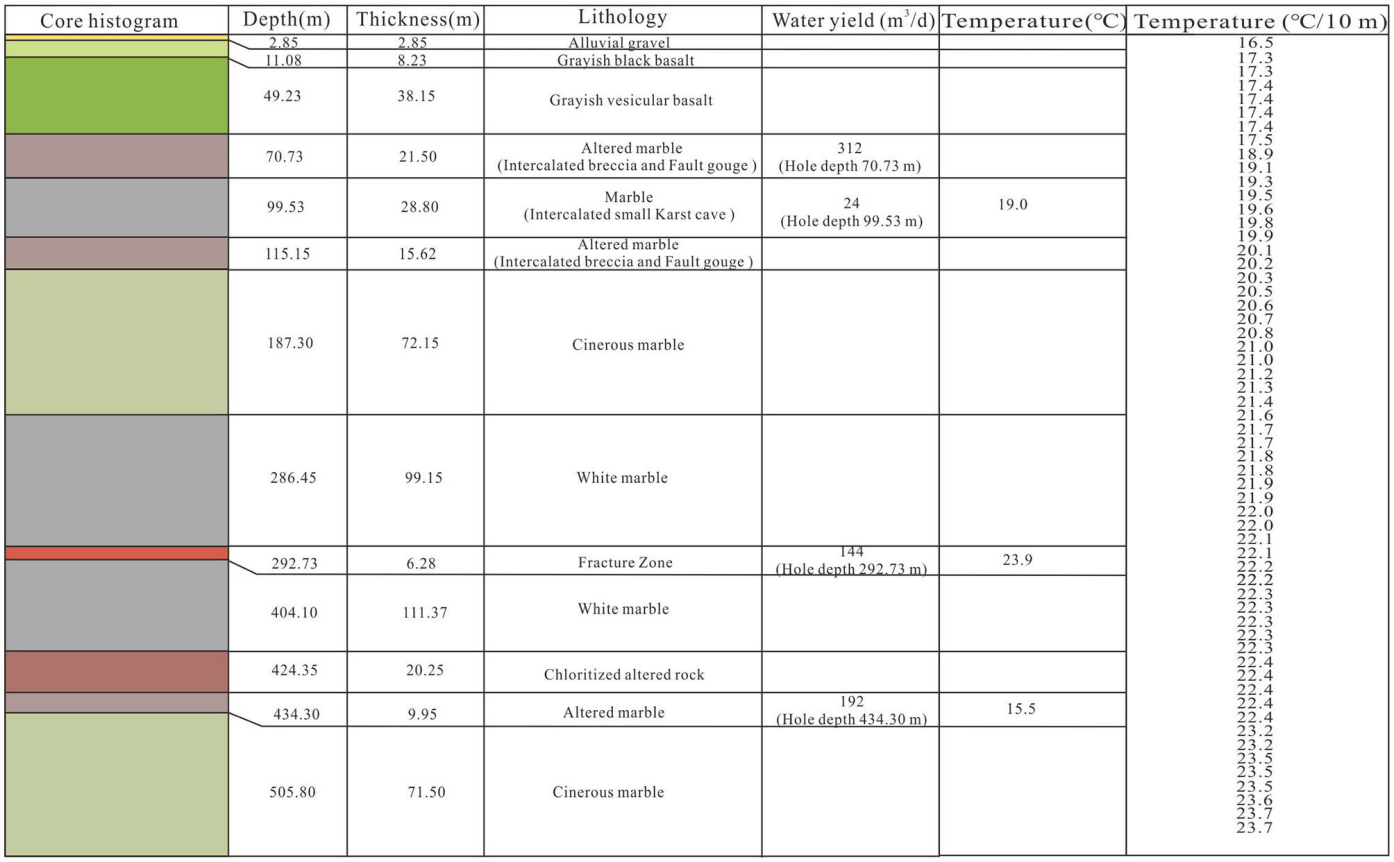
Lithology, water yield and temperature of geothermal well ZK1 in Erdaobaihe.

Effective assessment of potential of different types of geothermal resources is crucial and difficult. Results from the gravity planar overall indicate that the low gravity anomaly should be corresponding to the sedimentary basin and the high anomaly is related to the uplift mountain. Then, the advanced data processing technologies (upwards continuation and first vertical derivative) attempts to precisely assess geothermal resource potential in sedimentary basin and uplift mountain (Figure 9). In addition, based on the two point five dimensional man-machine interactive inversion technology, gravity sectional results indicate that the cataclastic marble and deep fault are geothermal reservoir and migration pathway of uplift mountain geothermal system, respectively. To the sedimentary basin geothermal system, pyroclastic rock is the primary geothermal reservoir and can migrate geothermal energy without fault. It is the formation thickness that determines the success of sedimentary basin geothermal system. Combing with regional geothermal well, temperature, lithology and water yield data, the geothermal resource in North Tianchi caldera can be catagorized into the sedimentary basin and uplift mountain geothermal system (unpublished data) (Figure 10).

## 6. Conclusions

(1) The two point five dimensional man-machine interactive inversion technology effective analyzed the four elements of deep geothermal resource in Changbaishan.

(2) Geothermal types in North Tianchi caldera and the periphery of North Tianchi caldera can be catagorized into the sedimentary basin and uplift mountain geothermal system, respectively.

## Data availability statement

The raw data supporting the conclusions of this article will be made available by the authors, without undue reservation.

## Author contributions

Z-HX and J-YJ designed carried out the surveys. Z-HX performed the geophysical data processing. G-WG and Z-JS made thematic figures. X-KJ and X-GN prepared the manuscript with contributions from all co-authors.
